# Cardiac Amyloidosis in Monoclonal Gammopathy of Undetermined Significance

**DOI:** 10.7759/cureus.118586

**Published:** 2026-10-25

**Authors:** Samuel Copen, Stephen Copen

**Affiliations:** 1 Biology, George Washington University, Washington, DC, USA; 2 Medicine/Nephrology, University of California, Los Angeles, Los Angeles, USA

**Keywords:** asymmetric septal hypertrophy, attr cardiac amyloidosis, cardiac mri, late gadolinium enhancement, late gadolinium enhancement (lge), monoclonal gammopathy of undetermined significance (mgus), mri cardiac, treatment of al cardiac amyloidosis

## Abstract

Patients with monoclonal gammopathy of undetermined significance are followed for years in hematology clinics, and most do not progress to develop defining events of multiple myeloma such as pathologic fractures or a high percentage of plasma cells on a bone marrow biopsy. Newer technologies such as cardiac MRI may lead to the definition of cardiovascular involvement by illness related to monoclonal gammopathy at an earlier time point than previously detected in clinical practice. The link between monoclonal gammopathy and an increased prevalence of transthyretin (ATTR) cardiac amyloidosis also confounds the question of whether changes detected on cardiac MRI indicate AL or ATTR cardiac amyloidosis. Here, a patient with both monoclonal gammopathy, cardiac amyloidosis, and a positive technetium pyrophosphate scan is referred for endomyocardial biopsy to clarify whether he suffers from AL or ATTR cardiac amyloidosis.

## Introduction

Patients with monoclonal gammopathy of undetermined significance are followed for years in hematology clinics, and most do not progress to develop defining events of multiple myeloma such as pathologic fractures or a high percentage of plasma cells on bone marrow biopsy. Additionally, 53% of myeloma cases are diagnosed five years after detection of monoclonal gammopathy, and clinical amyloidosis is diagnosed 93 months after onset of monoclonal gammopathy [1,2]. Newer technologies such as cardiac MRI may lead to the definition of cardiovascular involvement by illness related to monoclonal gammopathy at an earlier time point than previously detected in clinical practice [3]. The link between monoclonal gammopathy and an increased prevalence of transthyretin (ATTR) cardiac amyloidosis also confounds the question of whether changes detected on cardiac MRI indicate AL or ATTR cardiac amyloidosis.

AL amyloidosis refers to the deposition of excess immunoglobulins IgG, IgA, or IgM in the myocardium. ATTR amyloidosis refers to the deposition of tetramers of the thyroid hormone-transporting protein ATTR in the myocardium. MRI identifies the deposition of excess protein in the myocardium but cannot distinguish which protein is deposited in the myocardium. Here, a patient with diagnostic data suggesting both kinds of amyloidosis is presented, and an endomyocardial biopsy is required to distinguish which type of protein is deposited in the myocardium. Monoclonal gammopathy is present in this patient and predisposes to AL amyloidosis. However, the patient has had a technetium-99 hydroxymethylene diphosphonate scan that is characteristic of ATTR amyloidosis. Treatment depends upon making the correct diagnosis.

## Case presentation

A 73-year-old man had been followed in a nephrology clinic for 20 years for treatment of hypertension. He maintained good functional status, walking without dyspnea or other limitations. Examination over 20 years occasionally revealed minimal ankle edema. Blood pressure was usually controlled under 150/90 mmHg, most recently using metoprolol succinate 25 mg daily and telmisartan 40 mg daily.

His creatinine rose slowly from 1.2 mg/dL to 1.58 mg/dL. Twenty-four-hour urine protein was 565 mg. Serum protein electrophoresis revealed a monoclonal gammopathy with an M Spike of 0.51 g/dL. A renal biopsy (Figure 1) was performed and demonstrates hypertensive nephrosclerosis.

**Figure 1:**
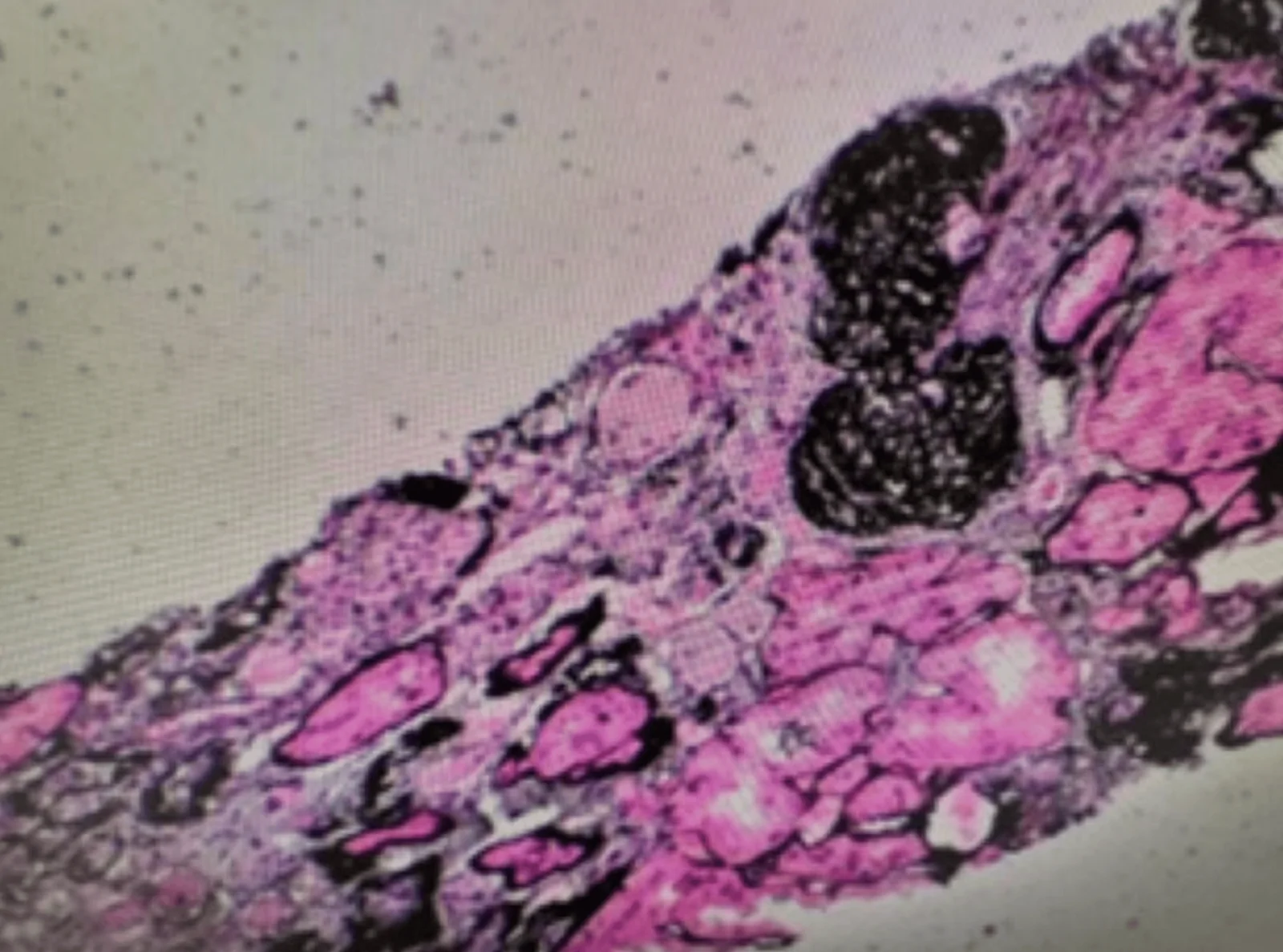
Renal biopsy in a patient with monoclonal gammopathy and 565 mg proteinuria Two globally sclerotic glomeruli, extensive tubulointerstitial scar, and severe arterial sclerosis.

The patient continued to be followed in the hematology clinic. He was diagnosed with IgG monoclonal gammopathy producing excess Lambda free light chains. Table 1 documents that over 22 months, the monoclonal spike rose to 0.5 g/dL and the kappa/lambda ratio declined from 0.27 to 0.15. A bone marrow biopsy showed three percent plasma cells. Figure 2 demonstrates that the lambda free light chains remain polyclonal on flow cytometry. 

**Table 1:** Hematologic, renal, and cardiac data IgG, lambda free light chains, M spike, creatinine, and brain natriuretic peptide increased over 24 months. Kappa/lambda free light chain ratio decreased over 24 months.

Parameter	Reference Range	Current	(-) 14 days	(-) 120 days	(-) 240 days	(-) 360 days	(-) 540 days	(-) 660 days	(-) 735 days	(-) 1680 days
IgG	700-1600 mg/dL	1618	1573	1742	1622	1476	1477	1467	-	-
IgA	76-426 mg/dL	206	216	237	218	192	1216	207	-	-
IgM	40-230 mg/dL	43	38	38	147	35	38	38	-	-
Kappa Quantative Free Light Chain	3.30-23.22 mg/L	44.34	47.47	41.38	37.59	34.04	38.06	42.04	-	-
Lambda Quanttative Free Light Chain	4.66-26.30 mg/L	292.24	282.73	243.17	215.16	197.18	173.65	158.37	-	-
Kappa/Lambda,Free	0.26-1.65	0.16	0.17	0.17	0.17	0.17	0.22	0.27	-	-
M Spike	zero g/dL	0.7	0.7	0.6	0.6	0.5	0.5	0.5	-	-
Creatinine	0.60-1.30 mg/dL	-	1.76	1.69	1.72	-	1.6	1.45	-	-
Brain Natriuretic Peptide	less than 100 pg/mL	289	-	-	-	-	-	-	220	20

**Figure 2:**
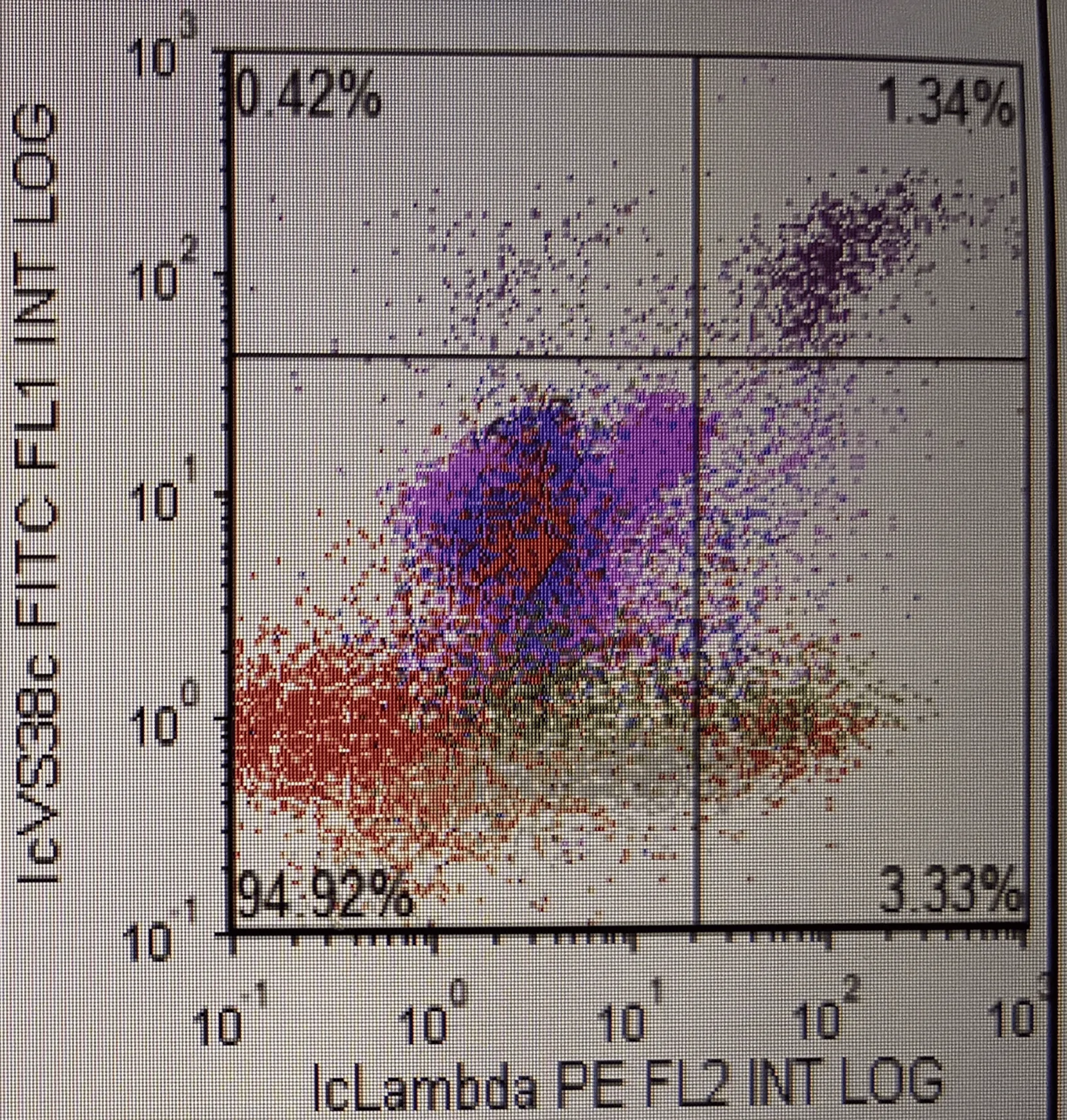
Flow cytometry Lambda free light chains are largely polyclonal, indicating a relatively low titer of monoclonal free light chains.

Figure 3 shows the echocardiogram, which demonstrated asymmetric septal hypertrophy, which has been associated with amyloidosis of the heart [4]. Left ventricular ejection fraction was between 65% and 70%. A mildly dilated right ventricle and moderately dilated left atrium were identified. Right ventricular systolic function was normal. Mild aortic regurgitation, mild mitral regurgitation, and mildly elevated pulmonary artery systolic pressure were identified

**Figure 3:**
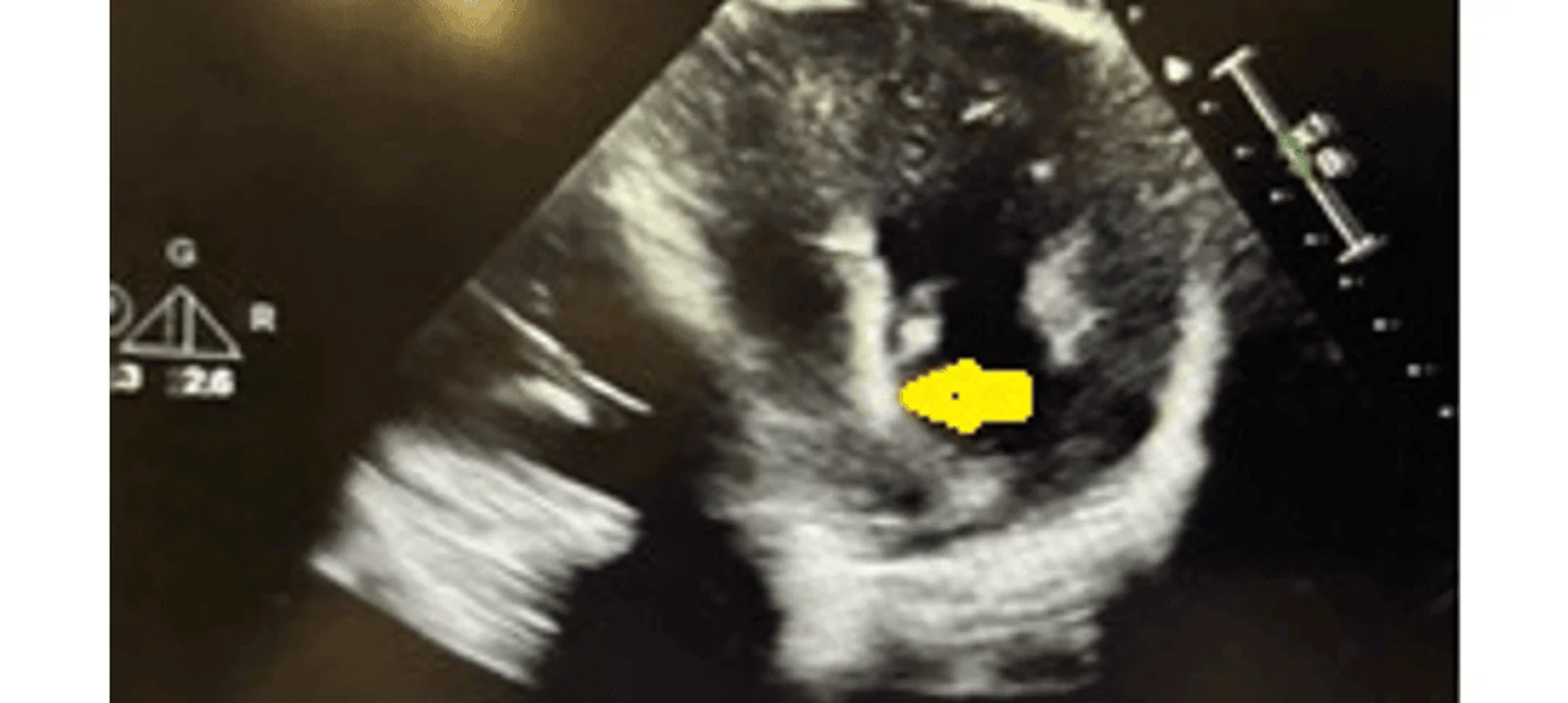
Echocardiogram demonstrating asymmetric hypertrophy of the interventricular septum The interventricular septal thickness during diastole was 1.8 cm, whereas normal is between 0.6 and 1.0 cm.

This warranted better definition with cardiac MRI shown in Figure 4. Diffuse patchy infiltration of the myocardium and late gadolinium enhancement are seen in cardiac amyloidosis as well as other cardiomyopathies and after myocardial infarction [5,6]. 

**Figure 4:**
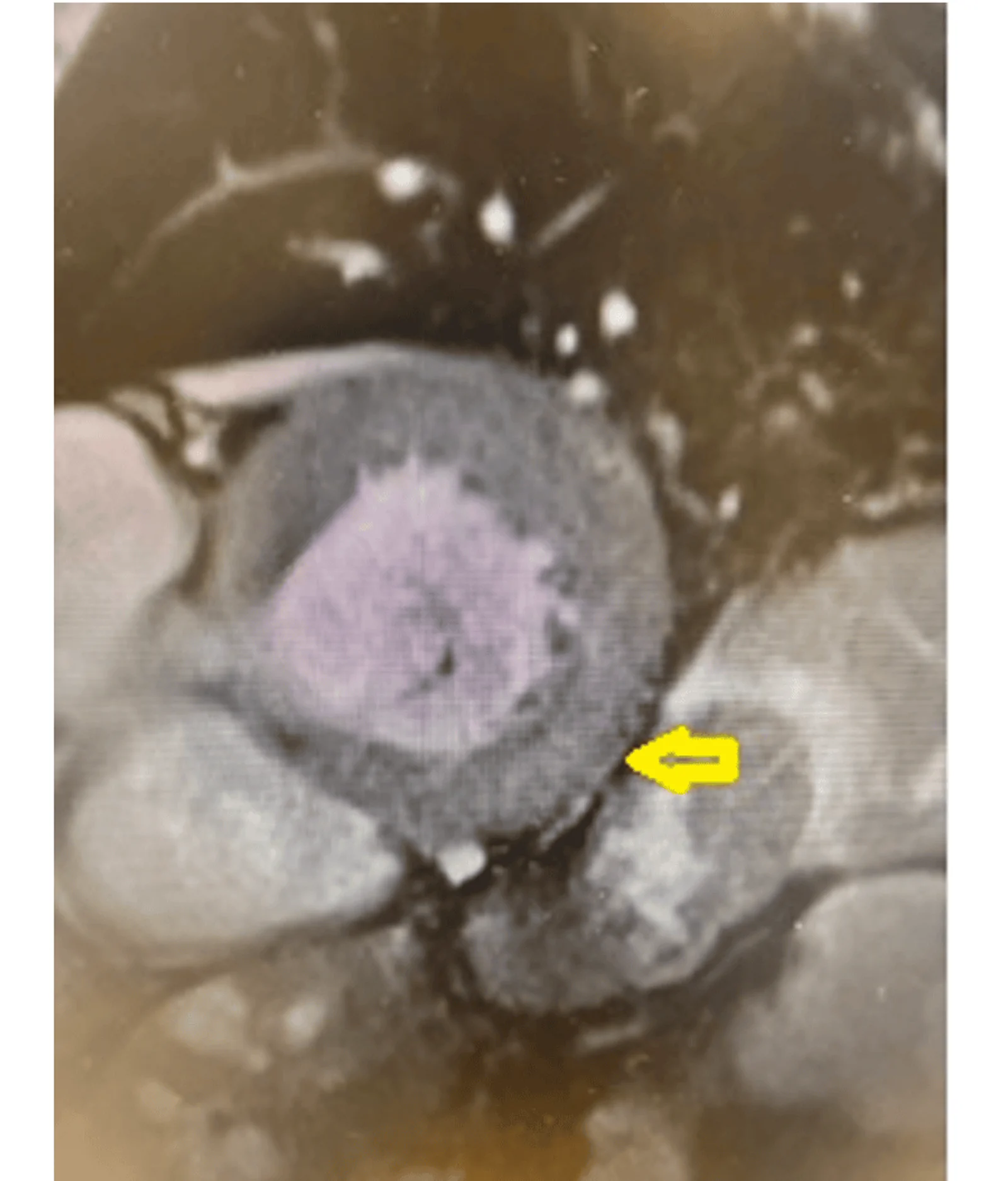
Cardiac MRI White enhancement in the left ventricular free wall surrounded by gray myocardium is characteristic of extensive diffuse patchy late gadolinium enhancement in a subendocardial-predominant distribution.

In the most recent nine months, the patient had an echocardiogram showing asymmetric septal hypertrophy, an MRI showing late gadolinium enhancement, and a technetium-hydroxymethylene diphosphonate scan. At this point, he was referred to a cardiac amyloidosis clinic, where the cardiologist recommended that the patient have an endomyocardial biopsy, which is pending.

Results of the technetium pyrophosphate scan shown in Figure 5 were not considered adequate to make a treatment decision [7,8].

**Figure 5:**
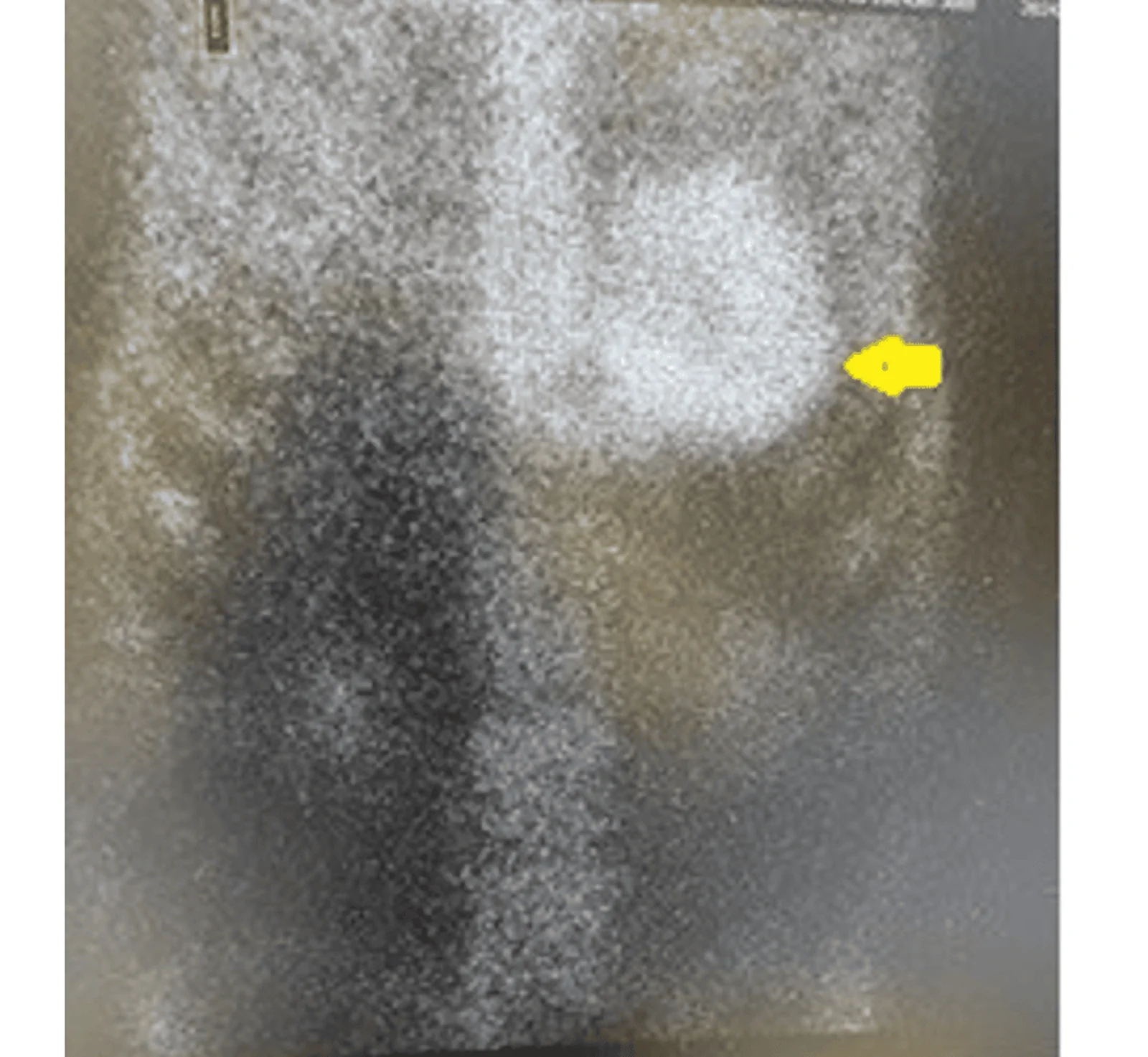
Technetium-99 hydroxymethylene diphosphonate scan Enhancement of the heart with Tc-99m hydroxymethylene diphosphonate, Grade 3 uptake.

When the endomyocardial biopsy is completed in the future, the specimen will be sent for laser dissection mass spectrometry, a technique which provides the highest specificity for diagnosis of the type of cardiac amyloidosis, AL versus ATTR [9].

## Discussion

Two types of cardiac amyloidosis exist: transthyretin amyloidosis and AL amyloidosis. Transthyretin-associated amyloidosis of the heart has been shown to occur in 23-49% of patients with monoclonal gammopathy of undetermined significance (MGUS), which is disproportionate to the occurrence of ATTR amyloidosis in the general population [10,11].

Patients with MGUS have also been documented to have AL amyloidosis [12-15].

Without treatment, patients with controlled symptoms of stage 1B ATTR cardiac amyloidosis have a median survival of 75 months. ATTR cardiac amyloidosis predisposes to atrial fibrillation and has improved prognostic outcomes upon treatment with tafamidis [16-19]. Treatment of ATTR cardiac amyloidosis leads to a 30% reduction in mortality at 30 months.

Without treatment, patients with stage 1, 2, and 3 AL cardiac amyloidosis have reduced survival [20-22]. AL cardiac amyloidosis also predisposes to arrhythmia and has been shown to have improved survival when treated with chemotherapy [1,23-27]. Treatment of AL cardiac amyloidosis is now associated with an increase in median survival to 55 months, with 22% of treated patients living 120 months.

## Conclusions

Here, a patient with hypertension and monoclonal gammopathy is found to have asymmetric septal hypertrophy on echocardiography. On magnetic resonance imaging, dense cardiac myocytes normally lead to rapid elimination of gadolinium from the myocardium, but infiltration of the myocardium by amyloid protein delays the elimination of gadolinium from the myocardium. This pattern of late gadolinium enhancement is a noninvasive, highly sensitive, and highly specific marker for cardiac amyloidosis.

In this patient with a monoclonal gammopathy raising the question of AL amyloidosis and a positive technetium-99 hydroxymethylene diphosphonate scan raising the question of ATTR amyloidosis, an endomyocardial biopsy is necessary to decide upon the correct treatment. At age 77, the patient chose to prioritize travel prior to consenting to have the endomyocardial biopsy, and his treatment will have to be deferred until this is completed.
